# Somatostatin Analogs in Clinical Practice: A Review

**DOI:** 10.3390/ijms21051682

**Published:** 2020-02-29

**Authors:** Mariana Gomes-Porras, Jersy Cárdenas-Salas, Cristina Álvarez-Escolá

**Affiliations:** 1 Department of Endocrinology, “La Paz” University Hospital. Paseo de la Castellana, 261, 28046 Madrid, Spain; marigomesp97@gmail.com; 2 Department of Endocrinology, “Fundación Jiménez-Diaz” University Hospital. Av. de los Reyes Católicos, 2, 28040 Madrid, Spain; jersy_cardenas@hotmail.com

**Keywords:** Somatostatin analog, pituitary adenoma, neuroendocrine tumor, cancer, carcinoid, lanreotide, octreotide, pasireotide

## Abstract

Somatostatin analogs are an invaluable therapeutic option in the diagnosis and treatment of somatotropinomas, thyrotropinomas, and functioning and non-functioning gastroenteropancreatic neuroendocrine tumors. They should also be considered an effective and safe therapeutic alternative to corticotropinomas, gonadotropinomas, and prolactinomas resistant to dopamine agonists. Somatostatin analogs have also shown to be useful in the treatment of other endocrine diseases (congenital hyperinsulinism, Graves’ orbitopathy, diabetic retinopathy, diabetic macular edema), non-endocrine tumors (breast, colon, prostate, lung, and hepatocellular), and digestive diseases (chronic refractory diarrhea, hepatorenal polycystosis, gastrointestinal hemorrhage, dumping syndrome, and intestinal fistula).

## 1. Introduction 

Somastostatin (SST) is a cyclic polypeptide derived from an SST precursor protein that is processed into several peptide hormones, including SST-14, SST-28, and neuronostatin. SST-14 is the isoform that was originally characterized [1]. STT is one of the main inhibitors of endocrine and exocrine hormone secretion in humans [2]. Native SST is not useful in clinical practice because it has an extremely short half life of 1–3 min, rapidly degradated by ubiquitously distributed peptidases in plasma and tissues [3]. After the characterization of SST, several SST synthetic analogs (SSAs) with longer half-life were developed. To date, three of them have been approved in clinical practice: lanreotide and octretide are considered first-generation SSAs, and pasireotide is considered a second-generation SSA. Their main clinical use has been evaluated in Phase III clinical Trials, and some other uses have been evaluated in prospective studies.

## 2. Physiological Actions of Somatostatin

**Figure 1:**
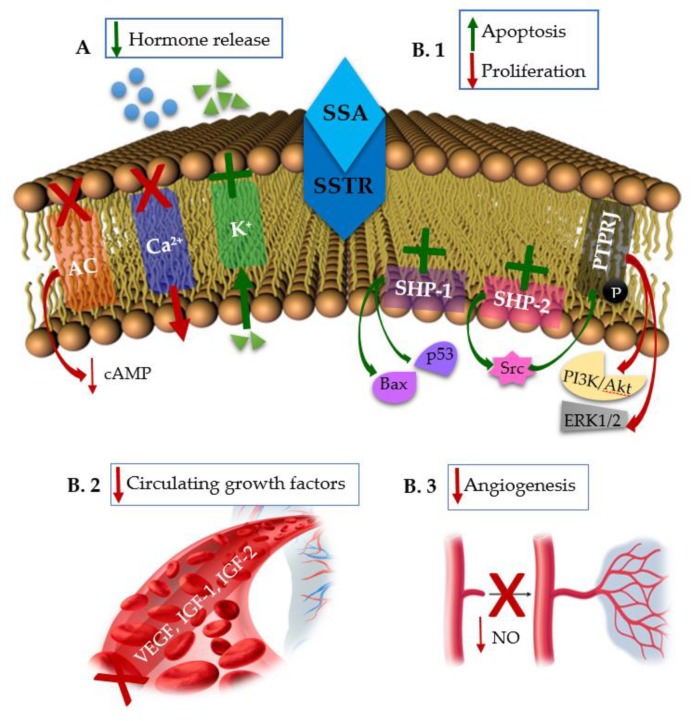
Mechanisms of action of Somatostatin synthetic Analogs (SSAs). (**A**). The antisecretory effects occur through the inhibition of the enzyme adenylyl cyclase (AC), the inhibition of voltage-dependent calcium channels and the stimulation of voltage-dependent potassium channels. (**B**). The antiproliferative effects. (**B.1**) Direct antiproliferative effects occur through the activation of the Src Homology 2 Domain Phosphatase-1 (SHP-1) and Src Homology 2 Domain Phosphatase-2 (SHP-2). SHP-1 triggers intracellular pro-apoptotic signals by the induction of p53 and Bax, while SHP-2 activates the tyrosine kinase Src that induces the phosphorylation of protein tyrosine phosphatase receptor type J (PTPRJ), which, in turn, dephosphorylates phosphatidylinositol 3-kinase (PI3K/Akt) and extracellular signal-regulated protein kinases 1 and 2 (ERK1/2), impairing cell proliferation. (**B.2** and **B.3**). Indirect antiproliferative effects occur through the inhibition of circulating growth factors like vascular endothelial growth factor (VEGF), insulin-like growth factor 1 (IGF-1), insulin-like growth factor 2 (IGF-2) (**B.2**) and through the inhibition of tumor angiogenesis by altering the release of nitric oxide (NO) (**B.3**). (Red “**X**”: Inhibition; Green “✚”: Stimulation).

STT inhibits the secretion of growth hormone (GH), prolactin (PRL), thyrotropin (TSH), cholecystokinin (CCK), gastric inhibitory peptide (GIP), gastrin, motilin, neurotensin, secretin, glucagon, insulin, and pancreatic polypeptide (PP). It also inhibits the exocrine secretion of amylase of salivary glands; hydrochloric acid, pepsinogen, and intrinsic factor of gastrointestinal mucosa; and enzymes and bicarbonate of pancreas and bile in the liver. Furthermore, glucose, fat, and amino acid absorption is inhibited by STT. STT also modulates gastrointestinal motility, delaying the late phase of gastric emptying, weakening gallbladder contraction, and prolonging small-intestinal transit time, but it also accelerates early gastric emptying and shortens the interval between migrating motor complexes. SST decreases portal (and varicose) pressure, inhibits retinal secretion and has an antidiuretic effect in humans. It is also believed that SST modulates activities of the central nervous system that underlie cognition and locomotion [4]. An inhibition of immunoglobulin synthesis and lymphocyte proliferation in lymphoid tissues has also been observed. An antiproliferative potential was also demonstrated by reversing the impact of mitogenic signals delivered by substances such as epidermal growth factor (EGF) and insulin-like growth factor 1 (IGF-1) [5,6]. The mechanisms of action of SSAs are described in Figure 1.

## 3. Somatostatin Receptors (SSTRs)

SST induces its biological effects by interacting with specific receptors that belong to the superfamily of G protein-coupled receptors. Five receptor subtypes have been identified (SSTR1–SSTR5), and the SSTR2 subtype has two splice variants (SSTR2A and SSTR2B). While all five subtypes are expressed in normal human tissues, the predominant subtypes in endocrine tissues are SSTR2 and SSTR5 [7,8,9]. Additionally, neuroendocrine neoplasms have repeatedly been shown to express SSTR2 and SSTR5 [10,11].

## 4. Somatostatin Analogs (SSAs)

The first synthetic SST analog (SSA) to be approved by the Food and Drug Administration (FDA) was the octapeptide octreotide (SMS 201-995) marketed as Sandostatin^®^ (50, 100, and 200 mcg subcutaneously every 8–12 h). It is available in both conventional and modified long-acting release (LAR) injection (Sandostatin LAR®), approved in 1988 and 1998, respectively. Sandostatin LAR® formulation contains octreotide distributed within polymer microspheres and it is available for intramuscular injection at doses of 10, 20, or 30 mg every 28 days [12]. Octreotide capsule, a novel orally-administered formulation that appears to be effective and safe for acromegaly treatment, is not yet approved for use and a new Phase III study is currently being conducted (clinicaltrials.gov: MPOWERED, NCT02685709) [13]. Octreotide shows high affinity for SSTR2 and inhibits the proliferation of the cells expressing *SSTR2* gene by activating the tyrosine phosphatase pathway [14].

Lanreotide is a cyclic octapeptide that was developed in the 1990s with the intent to develop a longer acting SSA. One of its initial formulations (BIM23014) had a half-life of 90 min [15]. A slow release formulation, lanreotide sustained release (lanreotide SR), followed soon after and has a half-life of 4.5 days. It was designed as microspheres of biodegradable polymers and it is available for intramuscular injection at doses of 30 or 60 mg every 7–14 days [16]. Some years later lanreotide Autogel® (ATG), a sustained release aqueous formulation, was presented as a prefilled syringe for subcutaneous administration at doses of 60, 90, or 120 mg every 28 days [17].

Lanreotide was approved for acromegaly treatment in Europe in the 1990s. The FDA approved lanreotide (Somatuline^®^ Depot) in the United States (US) for acromegaly treatment in 2007 for unresectable, well or moderately differentiated, locally advanced, or metastatic gastroenteropancreatic neuroendocrine tumors (GEP-NETs) in 2014 and for the treatment of Carcinoid Syndrome in 2017.

Pasireotide, a novel SSA, binds with higher affinity to SSTR1 (30-fold), SSTR3 (5-fold), and SSTR5 (39-fold), and with the same affinity to SST2 (three-fold) when compared with octreotide; and with higher affinity to SSTR1 (19-fold), SSTR3 (nine-fold), and SSTR5 (106-fold), but with the same affinity to SST2R (two-fold) when compared with lanreotide [18,19]. Pasireotide LAR (Signifor LAR^®^) was approved in 2014 by FDA and it is available for intramuscular injection at doses of 20, 40, or 60 mg every 28 days.

## 5. SSA in Clinical Practice

SST is a pluripotential hormone widely used in the treatment of multiple conditions, even outside the drug’s data sheet (off label). SSAs are mainly used for the diagnosis and treatment of well-differentiated GEP-NETs and in the treatment of acromegaly, as a first-line treatment or adjuvant after surgery. Because of their significant antisecretory, direct and indirect antiproliferative, and immunomodular activity, SSAs are also used in the field of endocrinology, oncology, digestive, general surgery, and ophthalmlology. (Table 1)

### 5.1. Pituitary Adenomas

The normal pituitary gland expresses a high proportion of SSTR5, lower levels of SSTR2, and very low levels of SSTR3 and SSTR1 [20]. SSTR4 is not expressed in healthy pituitary glands, nor in pituitary adenomas [21]. While SSTR3 is expressed in most pituitary adenomas, independent of the functional type, SSTR5 and SSTR2 (SSTR5 more than SSTR2) are widely expressed in somatotroph, thyrotroph, corticotroph, lactotroph, and gonadotroph adenomas and are probably involved in the regulation of hormonal secretion [22].

#### 5.1.1. Somatotropinomas

Acromegaly is a rare and severe systemic disease caused by GH-secreting pituitary adenoma (>75% are macroadenomas), which leads to hypersecretion of its peripheral mediator IGF-I. Transsphenoidal pituitary surgery is a first-line therapy, regardless of tumor size, with which biochemical control is obtained in approximately 75–90%, 40–50%, and 24% of the microadenomas, macroadenomas, and invasive tumors, respectively, depending on the learning curve and experience of the neurosurgeons [23,24,25].

Somatotropinomas predominantly express SSTR2 and SSTR5 (mainly SSTR2). SSAs are the treatment of choice when surgery is not curative, when it is not possible because the patient is not a candidate or when there is very low possibility of a complete surgical resection, and when the patient refuses it [26]. In addition to suppressing GH hypersecretion, significant tumor shrinkage has been seen after SSA treatment, particularly in patients undergoing primary medical therapy [27]. Higher SSTR2 expression improves the response to SSAs, while a lack of SSTR2 expression causes resistance. A high expression of SSTR5-TMD4, a truncated isoform, has been correlated with poor response to SSAs in somatotropinomas [28]. Female sex, elderly, lower circulating GH levels at diagnosis, somatic alpha-subunit of G stimulatory proteins (GSP) mutations, densely granulated pituitary adenomas, SSTR2A expression, and low Ki-67 index are predictors of greater sensitivity to SSAs [29]. However, aryl hydrocarbon receptor interacting protein (AIP) germline mutations or a low expression of AIP protein, sparsely granulated pituitary adenomas, and hyperintensity signal in magnetic T2 resonance images are associated with poor response to treatment with SSAs [29].

In the OASIS study, a multicenter observational study that included 74 adult patients with a recent diagnosis of acromegaly, surgery was the first therapeutic option in 76% of patients, persistent or recurrence disease was present in 73% after surgery, and in 90% of them, SSAs were used for disease control (54% as monotherapy), achieving a normalized IGF-1 level in 52% and disease control criteria in 47% of patients. In the remaining 24% of patients in whom surgery was not performed, SSAs were used as primary treatment, achieving disease control criteria in 22% of patients. Presurgical treatment with SSAs was used in 36% of patients and did not influence the surgical success rate [30]. Acromegaly treatment has changed over time, and surgery remains the first-line treatment; however, medical therapy has emerged as a new first-line treatment in some cases, SSAs being the most used. Radiotherapy rates have clearly declined [31,32].

The time needed to achieve normalization of GH and IGF-1 levels has also been studied; it was achieved after a median follow-up of 4.9 months (range 1.2–117) in 53 patients with active acromegaly under treatment with lanreotide ATG. Hormonal control was present in 64.9% of patients in less than 10 months [33].

If SSA therapy is discontinued, recurrence will be present in 56% of cases after 12–16 weeks of treatment withdrawal [34]. However, numerous cases and series of cases of prolonged remission and biochemical control of acromegaly after SSA withdrawal have been reported [35]. Remission is more likely in patients with a lower GH and IGF-1 level prior to treatment, absence of adenoma in magnetic resonance image (MRI) scans, with a long-term treatment and an adequate response to low doses. The rate of patients with prolonged remission (>12 months) varies highly from 16 to 41.7%, probably due to differences in inclusion criteria among the studies [35]. An expert consensus of acromegaly, published in 2014, recommends to consider treatment withdrawal in patients with well-controlled acromegaly, despite a decrease in SSA administration to the minimally effective dose and an increased dose interval (up to every 3 months). However, in these cases, lifelong IGF-1 levels should be monitored [32].

In patients with acromegaly that is not under control, despite high doses of first-generation SSAs, pasireotide LAR has been shown to provide superiority. The PAOLA study randomized 198 inadequately controlled acromegalic patients to receive pasireotide LAR 40 mg/28 days (*n* = 65), pasireotide LAR 60 mg/28 days (*n* = 65), and octreotide LAR 30 mg or lanreotide ATG 120 mg (*n* = 68), and found an adequate biochemical control in 15% and 20% of patients in the pasireotide LAR 40 mg and 60 mg groups, respectively; compared with no patients in the active control group. The two pasireotide LAR doses were not compared. Tumor volume reduction (≥25%) was achieved in 18.5% and 10.8% of patients who received 40 mg and 60 mg of pasireotide LAR, respectively. Improvement in quality of life was also better in the pasireotide LAR group, but hyperglycemia was more remarkable compared with active control [36]. In the 28-week extension of the PAOLA trial, biochemical control was maintained with pasireotide LAR and was generally well tolerated [37]. The superiority of pasireotide LAR in acromegalic patient’s resistant to first-generation SSAs could be related to the different SSTR expression profiles of the tumors and the higher affinity of pasireotide for SSTR 1, 2, 3, and 5. As mentioned before, low SSTR2 expression or a low SSTR2/SSTR5 ratio predicts resistance to first-generation SSAs. Pasireotide has shown to be more effective in lowering GH levels than octreotide in tumors with relatively high SSTR5 expression [38], and a higher SSTR5 expression has been found in somatotropinomas to be poorly responsive to octreotide [39].

Another treatment for acromegaly is pegvisomant, a highly effective GH receptor antagonist, used for treatment of patients that are intolerant or unresponsive/refractory to SSAs. The direct inhibition of GH action leads to a decrease in IGF-1 hepatic synthesis. Pegvisomant does not act directly on the adenoma, as the SSAs do; moreover, contrary to SSAs, it could increase GH secretion due to a loss of negative feedback from serum IGF-1 [40]. The efficacy of pegvisomant in achieving normal IGF-1 levels was up to 89% in clinical trials [41], while, in observational studies, it was reported to be of 62% and 73% after one and 10 years of treatment, respectively [40,42]. Combination treatment of pegvisomat and SSAs was proposed because of the different mechanism of action and potential synergistic effects, and it is only recommended in partial responders to SSAs [32]. It has proven to be highly effective in the normalization of IGF-1 levels in more than 90% of patients, to improve the quality of life, and to decrease tumor size in 20% of patients [43,44]. The advantages of a combination therapy are higher efficacy rates with lower doses of pegvisomant in the combined treatment than during pegvisomant monotherapy, tumor shrinkage, and cessation of tumor volume. Disadvantages include the increase in cost and side effects of both drugs. It was reported that pegvisomant serum levels increase by 20% when combined with SSAs [45]. Although pegvisomant action is not related to SSTR, it has been found that the required pegvisomant dose in combination with SSA therapy was inversely correlated with SSTR2 expression. This could be related to the lower SSTR2 expression at baseline in somatotropinomas resistant to SSAs, or because of feedback mechanisms of the GH–IGF-I axis and the downregulation of SSTR2 due to prior SSA treatment [46]. 

Presurgical treatment with SSAs is increasingly being used. It is proposed that presurgical treatment with SSAs is able to improve the general condition of the patient for surgery, to relieve the symptoms related to the disease, to reduce the risk of complications related to anesthesia (reduces laryngeal and pharyngeal soft tissue swelling and hypertrophy, to lower the frequency of cardiac arrhythmia, respiratory impairment, and respiratory infections), and to facilitate tumor resection by shrinking or softening pituitary adenomas (especially advantageous for invasive tumors), which would also result in better surgical outcomes. Presurgical treatment with lanreotide (3 months prior to surgery) was evaluated in a randomized trial that included 52 untreated patients with acromegaly, resulting from macroadenoma. A significant reduction in tumor volume, a change in tumor texture and lower invasion scores were observed before surgery in the lanreotide group. There was also a higher cure rate at 3 months after surgery in the lanreotide group (45.8%) than in control group (20%). Cured patients had greater tumor reduction during pretreatment [47]. Presurgical treatment with daily octreotide was assessed in 90 acromegalic patients with different sizes and invasiveness of adenomas (including microadenomas) and compared to 57 acromegalic patients who were not receiving octreotide treatment. Pretreatment achieved a tumor shrinkage in 31% of patients and tumors were observed to be slightly more often fluid or soft in texture (86% vs. 79%). The remission has a tendency to improve in the pretreatment group, but not statistically significantly so [48]. Octreotide pretreatment was also associated with shorter duration of hospitalization after surgery. Longer hospitalization was due to ventricular and supraventricular tachycardia in five patients, respiratory failure with sleep apnea in six patients, and respiratory infections in two patients [49]. A recent meta-analysis concluded that medical pretreatment does not increase the risk of postoperative complications, and leads to better short-term cure rates in acromegalic patients, but its impact on the long-term results is unclear [50].

#### 5.1.2. Thyrotropinomas

Thyrotropinoma is a very infrequent pituitary tumor, which represents 1% of pituitary adenomas, mostly macroadenomas, with locally aggressive behavior and clinical hyperthyroidism. The first therapeutic approach is transsphenoidal surgery or debulking, previous normalization of thyroid function, although it is curative in only 44% of cases [51,52]. Radiotherapy is used when remission is not achieved, but its effect is unpredictable and late, and the rate of remission is low [53]. 

SSAs constitute a treatment option for thyrotropinomas. In the presurgical stage, SSAs are effective in controlling hyperthyroidism and they improve surgery outcomes by reducing tumor size. As an adjuvant therapy to surgery, SSAs achieve euthyroidism in 75–95% of cases and a reduction in tumor size between 30–50% in the first 3 months of treatment [54,55,56]. For efficacy and safety, it should be noted that long-term treatment with SSAs should be maintained, at the lowest possible dose, to achieve biochemical and tumor control. 

#### 5.1.3. Corticotropinomas

Cushing’s disease appears when a corticotropin (ACTH) secreting pituitary adenoma is present. It represents 10% of pituitary adenomas. The treatment of choice is transsphenoidal surgery, which is curative in 50–90% of cases [57,58,59]. Radiation therapy and adrenolytic agents can be used as adjuvant treatment in case of incomplete surgical resections. 

Corticotropinomas express mostly SSTR5, followed by SSTR2. SSTR2 activation inhibits Proopiomelanocortin (POMC) synthesis, while SSTR5 activation inhibits cell proliferation. Corticotropinomas express almost exclusively high levels of POMC [60]. In an in vitro study, it was observed that octreotide exerted, in a shorter period of time, a greater degree of inhibition in a greater percentage of cells and managed to inhibit ACTH more than pasireotide [60]. However, another in vitro study in mice showed a more potent inhibition of ACTH with pasireotide compared to octreotide [19].

SSAs’ effects on ACTH secretion are less effective if cortisolemia is elevated due to SSTR downregulation by hypercortisolemia. Therefore, octreotide fails to suppress ACTH in most patients with untreated Cushing’s disease, while in Nelson syndrome, SSAs can inhibit ACTH secretion for a long time and even stabilize the tumor mass [61]. Therefore, the negative regulation of mRNA and SSTR2 levels by glucocorticoids may explain the lack of efficacy of SSAs in reducing ACTH and cortisol levels in patients with untreated Cushing’s disease [62].

The largest randomized phase III study to evaluate treatment with pasireotide in Cushing’s disease showed normalization of urinary free cortisol (UFC) after 6 months of treatment in 26.3% and 14.6% of the 162 patients randomized to receive subcutaneous injections of 900 µg and 600 µg twice a day, respectively. In addition, most patients experienced a reduction in UFC in the first two months, and the average reduction in UFC was 47.9% in both dose groups [63]. Eighty-one (54%) of the enrolled patients entered an extension study; the median overall exposure to pasireotide was 23.9 months and 39 (48.1%) patients completed the extension. Normal UFC was present in 51.9%, 16.0%, and 53.1% at extension baseline, 36th month, and last assessment, respectively. Diabetes was present in 81.5% and 88.9% at extension baseline and last assessment, respectively [64].

The use of SSAs has also been proposed to make a differential diagnosis of Cushing’s disease from ectopic ACTH secretion, because SSAs are effective in inhibiting ACTH and cortisol in ectopic tumors [65]. The location of the ectopic tumor is usually difficult, thus OctreoScan^®^ and Ga-68 DOTATE allow to locate the ectopic tumors producing ACTH, CRH, and their metastases [66].

#### 5.1.4. Gonadotropinomas

Gonadotropinomas are pituitary tumors that produce intact gonadotrophins and/or their α and β subunits. Approximately 80–90% of non-functioning pituitary tumors are gonadotropinomas, which account for as many as 40–50% of all pituitary macroadenomas [67]. The treatment of choice is transsphenoidal surgery, with radiotherapy as an adjuvant treatment. Although gonadotropinomas have been shown to express SSTR (mainly SSTR2 and SSTR5) and type 2 dopamine receptors (DR2), the results with SSAs and dopamine agonists (DAs) are discrete regarding tumor shrinkage [67]. The limited available data indicate that SSAs achieve a significant reduction in tumor size in only 12% of patients [68,69,70,71]. More recently, the results of the PASSION-I phase 2 trial have shown that a reduction in size of at least 20% was achieved in only 16.7% of patients after pasireotide LAR treatment [72].

The combination of SSAs and DAs is able to inhibit the secretion of gonadotropins in vivo and in vitro [73], reduce tumor size by 20% after 12 months of treatment [74], and achieve a campimetric improvement of 33–50%, probably by cerebral vasodilator effect of SSAs [71,75]. It has been reported that 12-month treatment with SSAs and DAs controls the tumor in 27% of cases [74]. SSAs and DAs could be a treatment option in aggressive gonadotropinomas in which pituitary surgery is contraindicated or is not curative, as an adjunctive treatment to radiotherapy [76].

#### 5.1.5. Prolactinomas

Prolactinomas are the most frequent subtype of adenoma and occur in approximately 30% of patients with multiple endocrine neoplasia type 1 (MEN-1). They are more frequent in women (sex ratio 2:1), who usually have microadenomas; while in men, due to diagnostic delay, they are usually macroadenomas [77]. For both, micro- and macroprolactinomas, DAs are the treatment of choice. Cabergoline is the most effective DA available; it normalizes PRL levels, reduces tumor size, and restores gonadal function in 95% and 80% of patients with microprolactinomas and macroprolactinomas, respectively [78,79,80,81]. Dopamine agonist-resistant prolactinomas (DARPs) are more prevalent in young men with cystic tumors and are generally macroadenomas with cavernous sinus invasion [82]. Surgical debulking of the tumor, radiotherapy, and/or unconventional medical treatment (SSAs, temozolomide, or tamoxifen) are treatment options in DARPs [83]. Thus far, temozolomide treatment in 15 cases of malignant prolactinomas has shown complete response in two patients and a partial response in seven [84]. SSTR expression in prolactinomas is very variable with predominance of the SSTR5 [21]. Studies with first-generation SSAs have not demonstrated efficacy in DARPs, and in vitro studies have reported inconsistent results, probably due to differences in SSTR2 expression, since transference of the *SSTR2* gene to prolactinomas make them sensitive to octreotide [85,86]. Some case reports have shown that pasireotide LAR is able to normalize PRL levels and control symptoms after one month of treatment, inducing necrosis of the tumor cells and/or cystic degeneration [87].

### 5.2. Gastroenteropancreatic Neuroendocrine Tumors (GEP-NETs)

Neuroendocrine tumors (NETs) are a heterogeneous group of tumors that originate in neuroendocrine cells from endocrine glands, endocrine islets, and endocrine cells dispersed throughout the digestive and respiratory tract. The gastrointestinal tract (62–67%) and the lungs (22–27%) are the most common primary tumor sites and the majority are sporadic [88]. GEP-NETs commonly express SSTR [88]. Approximately 80% of NETs of the gastrointestinal tract express SSTR2 [89]. In fact, in patients with GEP-NETs treated with SSAs, expression of SSTR2 is associated with longer progression-free survival (PFS) [90].

#### 5.2.1. Functioning Pancreatic NETs

Pancreatic-NETs express SSTR1, 2, 3, and 5. Insulinomas have a heterogeneous expression of STTR, while all somatostatinomas express SSTR5 and all gastrinomas and glucagonomas express SSTR2 [91]. SSAs are the standard therapy in well-differentiated, locally advanced, or metastatic GEP-NETs of any size [92]. The efficacy of octreotide LAR and lanreotide ATG has not been directly compared; however, due to its biochemical similarity, it is assumed that both are equivalent in the control of symptomatic GEP-NETs, as recommended by the guidelines of the National Comprehensive Care Network (NCCN) [93]. 

Insulinomas: SSAs are the second-line medical treatment for controlling hypoglycemia in patients with insulinomas, mainly for malignant insulinomas. Octreotide was effective in controlling hypoglycemia in 59% of patients with insulinoma [94]. Pasireotide is able to control hypoglycemia in insulinomas resistant to other treatment options (octreotide LAR, everolimus, chemotherapy) and may be an alternative therapeutic option in malignant insulinomas and recurrent hypoglycemic events [95]. However, in insulinomas without SSTR expression, SSAs worsen hypoglycemia by inhibiting counter-regulatory mechanisms (glucagon and GH) [96]. 

Gastrinomas: High doses of proton pump inhibitors are effective in reducing hypersecretion of gastric acid, but do not reduce hyperplasia of enterochromaffin-like (ECL) cells. In contrast, several studies with SSAs (octreotide LAR, lanreotide) in gastric NETs type 1 (associated with chronic atrophic gastritis) and type 2 (associated with Zollinger-Ellison syndrome) have been shown to inhibit gastrin secretion (decreases or normalizes gastric secretion in 50–100% of gastrinomas) and to reduce tumor burden, achieving tumor stabilization in 47–75% of patients. Moreover, SSAs may prevent ECL cell hyperplasia or the development of gastric NETs type 2 [97,98,99].

Glucagonomas: In glucagonomas, octreotide and lanreotide have shown rapid relief of migratory necrolytic erythema and diarrhea, despite the persistence of elevated serum glucagon levels [100,101,102], while pasireotide has been suggested as an appropriate therapeutic option in first-generation SSA-resistant glucagonomas.

Vipomas: In the case of Vipomas, adjuvant octreotide treatment was effective in controlling diarrhea and reducing serum VIP levels [103,104,105].

Somatostatinomas: Although it seems paradoxical to treat somatostatinoma with SSAs, octreotide improved the related symptoms and reduced SST plasma levels in three patients [106].

#### 5.2.2. Antiproliferative Effects in GEP-NETs

Although SSAs were developed primarily for symptomatic control of NETs (antisecretory effect), the results of the PROMID, CLARINET, and CLARINET OLE studies confirmed that SSAs also have a positive impact on the progression-free survival (PFS) in pancreatic and non-pancreatic NETs due to their antiproliferative effects [107,108,109].

The results of the PROMID study ratified the antitumor effect of SSAs and generalized the use of octreotide LAR in the treatment of NETs, regardless of their functionality. The study included 85 treatment-naive patients with well-differentiated (Ki67 < 2%) locally inoperable or metastatic midgut NETs (functioning and non-functioning) and they were randomly assigned to either octreotide LAR (30 mg monthly intramuscularly) or placebo for 18 months until tumor progression or death. After 6 months of treatment, in the octreotide LAR group, the disease remained stable in 66.7% of patients compared to 37.2% of patients in the placebo group. PFS was significantly greater in the octreotide LAR group compared to placebo (14.3 months vs. 6 months, respectively; HR = 0.34). The beneficial effect of octreotide LAR on overall survival could not be established [107].

The CLARINET study included 204 treatment-naive patients with advanced well or moderately differentiated (Ki67 < 10%) non-functioning GEP-NETs, including pancreatic and gastrointestinal tumors, and they were randomly assigned to either lanreotide ATG (120 mg/28 days) or placebo for 96 weeks. The median PFS was not reached for lanreotide ATG in the 96-week study compared to 18 months with placebo (HR = 0.47). The rates of PFS were 65.1% the lanreotide group and 33.0% in the placebo group at 24 months. There were no differences in quality of life or overall survival [108].

The CLARINET OLE study was an extension study that further investigated the safety and efficacy of lanreotide ATG in patients with metastatic pancreatic and nonpancreatic NETs. Eighty-eight patients with stable disease were selected, 41 continued with lanreotide ATG and 47 patients switched from placebo to lanreotide ATG (120 mg/28 days). The median PFS was 32.8 months for patients originally randomized to lanreotide ATG in the central study compared with the 18 months for placebo reported in the CLARINET study [108]. The most common adverse effect was diarrhea. However, among the patients who received lanreotide in both studies, diarrhea improved with greater exposure to treatment [109].

Pasireotide LAR 60 mg demonstrated a higher antitumor effect than octreotide LAR 40 mg in a phase III trial that included 216 patients with metastatic GEP-NETs who had inadequately controlled carcinoid symptoms, with a higher median PFS (11.8 months vs. 6.8 months) and higher tumor control rate at month 6 (62.7% vs. 46.2%, respectively), although statistically, it was not significant (*p* = 0.09) [110]. In a phase I, dose escalation study, pasireotide concentrations showed a direct relationship with tumor shrinkage and an inverse relationship with IGF-1 levels. The two radiologically objective partial responses occurred in the 120 mg group; this group also showed a higher incidence of bradycardia (<40 bpm) [111].

Primary pancreatic tumor, distant extrahepatic metastases, and non-resection of the primary were identified as predictive factors of negative response to SSAs [112]. Ki67 index <5%, tumor stability, and a hepatic tumor load <25% involvement were predictors of a positive response to SSAs [113]. The response of octreotide LAR in the PROMID study was higher if the primary tumor was resected and if the liver tumor load was ≤10% [107].

In 2016, a large retrospective study that included 254 patients determined the predictive factors of radiological response to octreotide in advanced NETs. Most patients had small bowel primary tumors (*n* = 204), followed by pancreatic (*n* = 22), lung (*n* = 14) and rectal primaries (*n* = 7). Pancreatic, rectal, and pulmonary primary tumors, G2, liver metastases, and Chromogranin A (CgA) >10 times the upper limit of normality were associated with an unfavorable response to SSAs. In men, asymptomatic patients with stable disease and carcinoid heart disease were favorable predictors of tumor response to SSAs. In this study, age, functional status of the tumor, primary tumor resection, extrahepatic metastases, and mesenteric desmoplasia did not seem to have any effect on treatment with SSAs [114].

The retrospective GETNE-TRASGU study evaluated 535 patients with advanced well-differentiated (Ki-67 ≤ 20%) GEP-NETs (pancreas, GI, or unknown origin) treated with SSAs alone. Median PFS was 28.7 months and overall survival was 85.9 months. The significant variables associated with PFS were presence of symptoms when initiating treatment, primary tumor location, Ki-67, extent of liver involvement, presence of bone and peritoneal metastases, documented progression status, neutrophil-to-lymphocyte ratio, and alkaline phosphatase. The GETNE-TRASGU model could be a valuable tool for making treatment decisions in daily clinical practice [115].

Non-conventional SSA doses include higher administered doses or decreased dose interval. In 2019, a multicenter Italian study with non-conventional doses of SSAs (5% with lanreotide 180 mg or octreotide LAR 60 mg every 28 days, or 95% with lanreotide 120 mg or octreotide LAR 30 mg every 14–21 days) found that the administration of unconventional doses of SSAs as earlier line of treatment is associated with increased PFS and lower risk of progression or death. It included 140 patients, previously treated with SSAs, with progressing well-differentiated GEP-NETs. No statistically significant differences were observed in PFS according to sex, primary tumor site, disease extension, liver metastases, tumor primary surgery, WHO 2010 classification, presence of hormonal hypersecretion syndrome, or type of unconventional SSA dose. Higher risk of progression or death was associated with third or further lines of treatment compared to second line (HR = 1.95). Treatment with non-conventional doses was safe and well tolerated. Side effects were: G1–2 worsening of serum glucose level control (19 events), G1 diarrhea (16 events), G1 fatigue (3 events), and G3 cholelithiasis (two events) [116].

#### 5.2.3. Carcinoid Syndrome

Carcinoid syndrome includes a set of signs and symptoms derived from the release of vasoactive amines and prostaglandins not metabolized to systemic circulation by a carcinoid tumor. Carcinoid crisis can occur spontaneously or can be caused by physical stress and/or psychological, alcohol, tyramine-rich foods, or anesthesia [117]. The most common manifestation (85%) is the sudden flushing of the face, neck, and upper thorax associated with heat sensation, pruritus, tachycardia, and arterial hypotension. As the disease progresses, the episodes of flushing are more intense and frequent, being able to permanently acquire a pinkish coloration with telangiectasias. Diarrhea and abdominal pain manifest in 80% of cases and may present with specific symptoms of flushing episodes. Bronchospasm and dyspnea occur less frequently (15%), often coinciding with episodes of flushing. Carcinoid heart disease is characterized by fibrotic lesions mainly in the right heart. Other manifestations are pellagra, weakness, and muscular atrophy [118]. 

The pooled results of 11 studies with lanreotide were comparable with those of octreotide, obtaining a decrease in diarrhea in 75% and flushing in 80% of cases [119]. Similarly, in a multicenter and cross-sectional study that included 33 patients with carcinoid syndrome, half of them received daily octreotide for a month, followed by lanreotide every 10 days for 1 month, while the other half commenced with lanreotide, followed by octreotide. Both were equally effective in reducing symptoms, although patients preferred the lanreotide treatment scheme [120]. With respect to depot formulation, the results with lanreotide ATG (*n* = 55 patients, 120 mg/month) were comparable with other lanreotide preparations, showing a resolution of 75% of diarrhea and 80% of flushing [121]. Similarly, in the ELECT study, 150 patients with carcinoid syndrome were randomized to lanreotide depot (120 mg) or placebo every 4 weeks, with access to octreotide rescue, and it was observed that lanreotide depot reduced the use of rescues by 15% (*p* = 0.036) and controlled diarrhea and flushing by 76% and 73%, respectively [122]. The SymNET study included 340 patients with a history of carcinoid syndrome-related diarrhea on treatment with lanreotide ATG (120 mg/month) for more than 3 months. The study showed that 76% and 73% of patients were satisfied with diarrhea and flushing control, respectively [123]. In cases of refractory carcinoid syndrome, treatment options include increasing the dose of SSAs, shortening the interval between one dose and the next, or supplementing the long-acting formulation with a short-acting formulation. In one phase II multicenter study in 45 patients with metastatic carcinoid tumors refractory/resistant to octreotide LAR, it was shown that pasireotide (60–120 mg administered subcutaneously twice daily) was effective in controlling noncardiac symptoms (diarrhea/flushing) in 12 patients (27%); three of these patients achieved complete control of symptoms, while nine experienced partial control [124]. However, in one phase III multicenter study that included 110 patients with refractory carcinoid symptoms, it was not shown that pasireotide (60 mg/28 days) was superior than octreotide LAR (40 mg/28 days) in symptom control after 6 months of treatment [110]. Usually, carcinoid syndrome occurs when the tumor has metastasized, often to the liver, and the bioactive substances reach systemic circulation in high concentrations. A prospective and multicenter study with a parallel design randomized 93 patients with malignant carcinoid syndrome to receive 10 mg (*n* = 22 patients), 20 mg (*n* = 20 patients), or 30 mg (*n* = 25 patients) of octreotide LAR or octreotide (*n* = 26 patients) every 8 h and demonstrated that both formulations achieved similar control of symptoms, though they recommended an initial dose of 20 mg of octreotide LAR. The authors highlighted the need for subcutaneous doses of octreotide for approximately 2 weeks after the start of octreotide LAR, and even rescued a dose for 2 to 3 months until steady-state octreotide levels from the LAR formulation were achieved [125]. In an expert statement on the diagnosis and treatment of carcinoid heart disease in patients with neuroendocrine tumors, long-acting SSAs are recommended to prevent its development and/or progression [126]. Short-acting formulations are especially useful in preventing and treating perioperative carcinoid crisis and in the management of carcinoid crisis, and may have a role as rescue medication in refractory carcinoid syndrome. NCCN guidelines on NET specifically state that octreotide should be initiated in all patients with functional carcinoid tumor before surgical resection of the primary tumor and/or its metastases. Although an ideal scheme and dosage of octreotide has not been established to prevent carcinoid crisis in the perioperative period, several schemes have been proposed depending on the type of surgery and the individual risk of developing a carcinoid crisis [93].

#### 5.2.4. GEP-NETs in Multiple Endocrine Neoplasia Type 1 (MEN-1)

MEN-1 is an autosomal dominant hereditary syndrome characterized by the development of multiple NETs and/or hyperplasia, involving mainly duodenum and pancreas, parathyroid glands, and the anterior pituitary gland. The basis of the treatment of NETs in MEN-1 is surgery, but it is associated with a high risk of complications, mortality, and high recurrence rate. In patients with MEN1-related non-functional NETs <20 mm, surgery or medical treatment is not recommended [127]; however, some studies have proven SSAs to be effective in controlling the tumor [128].

A retrospective study evaluated the efficacy of octreotide LAR (30 mg/month) for 12–75 months as a first-line treatment in 20 patients with one or more early-stage duodeno-pancreatic NETs (70% non-functioning, 25% Zollinger-Ellison syndrome, and 5% insulinomas) <20 mm related to MEN-1 and demonstrated an objective tumor response in two (10%) patients (both affected with a non-functioning NETs) and stable disease in 16 (80%) patients (12 with non-functioning NETs and four with a gastrin-secreting NETs). In a biochemical evaluation, five patients (25%) achieved prolonged normalization over time of CgA and/or gastrin levels. Moreover, the clinical symptoms related with Zollinger-Ellison syndrome and insulinoma were resolved in all patients and one patient, respectively [128].

In 2019, the first prospective study was conducted to evaluate the effectiveness of lanreotide ATG (120 mg every 28 days) for 24–84 months in 23 patients with one or more MEN1-related NETs <20 mm of maximal diameter. The median progression-free survival was significantly higher in the lanreotide ATG than in the active surveillance group (median not reached vs. 40 months). In addition, in the lanreotide group, 17.4% of patients had an objective tumor response, 35.5% of patients had a variable degree of tumor shrinkage, 65.2% patients had a stable disease, and 100% of patients normalized baseline CgA levels. Only three patients in the lanreotide ATG group experienced mild adverse effects (G1 diarrhea and asymptomatic cholelithiasis) [129].

Therefore, there have been reports of patients with MEN-1 with multiple GEP-NETs (non-functional, insulinomas, Zollinger-Ellison syndrome) in concomitant treatment with SSAs and DAs that presented initial clinical improvement and complete and stable clinical remission. Therefore, a possible synergistic effect of SSAs and DAs is proposed, particularly in MEN-1, to induce antitumor effects and to normalize the functional syndrome [130]. 

### 5.3. Other Diseases

#### 5.3.1. Congenital Hyperinsulinism (CHI)/Persistent Hyperinsulinemic Hypoglycemia of Infancy

Congenital hyperinsulinism is characterized by hypoglycemic state secondary to inappropriately elevated insulin levels. CHI is responsible for most cases of persistent hyperinsulinemic hypoglycemia in newborns. It can arise sporadically or can be inherited in an autosomal recessive way (more frequently by mutations in the *ABCC8* and *KCNJ11* genes), and can affect the pancreas in a focal, diffuse, or atypical way. Severe and/or persistent hypoglycemia produces irreversible neurological alterations and severe retardation of psychomotor development [131]. 

The only drug approved to treat CHI in childhood is diazoxide. However, since 1986, SSAs have been used in the treatment of patients with CHI who do not respond to diazoxide, being considered a second line treatment in CHI [132], over nifedipine or continuous glucagon. SSAs are potent inhibitors of insulin and glucagon secretion by activating SSTR (mainly SSTR2). Octreotide inhibits insulin secretion by binding to SSTR2 and SSTR5. Activation of SSTR5 decreases insulin gene promoter activity, and inhibits calcium mobilization and acetylcholine activity. SST also inhibits the ATP-sensitive potassium (KATP) channel, which results in reduced insulin secretion. In a large series, octreotide was found to be a safe and effective treatment for diazoxide-unresponsive CHI patients. The effects on linear growth were found to be clinically insignificant. Monthly injections of long-acting SSAs have been described as an effective option in the management of CHI. Octreotide LAR and lanreotide ATG have been used successfully in children with CHI, even in early infancy. Using long-acting formulations increases treatment adherence and improves quality of life (QoL) [133]. 

#### 5.3.2. Diabetic Retinopathy (DR) and Diabetic Macular Edema (DME)

Although the treatment of choice for diabetic retinopathy (DR) is laser photocoagulation, when the diagnosis is delayed, its evolution is usually torpid. In these cases, intravitreal injection of SSAs is a promising therapeutic alternative for the control of proliferative DR and frequently diabetic macular edema (DME). By combining laser photocoagulation with SSAs, a more powerful and lasting effect is obtained to help stabilize or stop visual impairment [134].

The human retina, especially the pigment epithelium layer, has abundant SST and SSTR [135]. It has been determined that there is a lower concentration of SST in patients with DR and DME compared to controls [136]. The low intravitreal levels of SST in patients with proliferative DR and DME contribute to angiogenesis in response to ischemia characteristic of the proliferative form of DR, as well as to the disruption of the blood–retina barrier involved in the pathogenesis of DME. Experimental studies have shown that SSAs exert a neuromodulator and antiangiogenic effect on the retina, preventing hemorrhagic and proliferative phenomena and improving the integrity of the blood–retina barrier [137].

In addition, GH and IGF-1 levels play an important role in the pathogenesis of DR. GH inhibition is another beneficial effect of SSAs on DR [138,139]. A reduction in the risk of hemorrhages and other retinal vitreous complications has been demonstrated in diabetics with proliferative DR on treatment with SSAs [134,140,141].

#### 5.3.3. Graves’ Orbitopathy

Graves’ orbitopathy is an extrathyroid manifestation of autoimmune hyperthyroidism. SSTR is present in extraocular muscles, lymphocytes, and fibroblasts (Graves’ disease target cells), as well as immunoreactivity for IGF-1 in extraocular muscles and adipocytes [142]. Thus, SSAs act on the triggers and perpetuators of the disease by inhibiting cytokines (TNF-α, IFN-γ, IL-1, TGF-3) and growth factors (mainly IGF-1) [143].

In vivo studies have demonstrated a relationship between the presence of SSTR and the activity of thyroid orbitopathy, determined by OctreoScan^®^ [144,145]. However, it remains to be determined whether OctreoScan^®^ is a parameter of disease activity and a predictor of immunosuppressive therapy outcome better than clinical parameters, MRI, or measurement of glycosaminoglycan in plasma and/or urine.

Several studies in small cohorts and without a control group have shown that octreotide and lanreotide improve symptoms (Soft tissue inflammation, palpebral aperture, intraocular pressure, exophthalmos, diplopia, cornea, and visual acuity), inactivate the disease at 3 months, and reduce the use of systemic corticosteroids. Therefore, SSAs are a diagnostic, therapeutic, and predictive option of the therapeutic response to immunosuppressants of Graves’ orbitopathy in active phase [146,147,148,149].

#### 5.3.4. Dumping Syndrome

The dumping syndrome is a set of gastrointestinal and vasomotor symptoms. It results from changes in the anatomy and physiology of the stomach. It is a frequent complication of gastric surgery (5–10%), although its frequency is higher after vagotomy with pyloroplasty (20%) and esophagectomy (50%). Dumping syndrome has two phases; early dumping occurs within 30 min after a meal and is due to the rapid transit of hyperosmolar food into the upper small bowel, while late dumping often occurs 2–3 h postprandially and is due to hypoglycemia attributed to hyperinsulinemia secondary to the rapid initial rise of blood glucose concentration [150]. 

Most patients with mild dumping syndrome feel better after dietary modification (particularly the reduction of carbohydrate intake), and it is considered the first-line approach. However, severe dumping symptoms (5–10%) may continue despite dietary changes [151]. SSAs may play a role in the pathophysiology of dumping syndrome because they can retard gastric emptying rate, retard transit through the small bowel, inhibit the vasoactive intestinal polypeptide, gastric inhibitory peptide, and insulin release, inhibit postprandial vasodilation and splanchnic vasoconstriction, and increase intestinal absorption of water and sodium [152]. 

Some studies have shown that SSAs relieve symptoms both of early and late dumping syndrome, although the benefit is greater in the former, particularly in postprandial tachycardia [153,154]. Octreotide decreases cardiac frequency, the Sigstad index score, and plasma insulin levels, and minimizes changes in orthostatic blood pressure, packed cell volume, and plasma osmolarity in subjects with dumping [155]. In the long term, octreotide has been shown to maintain its efficacy in patients with refractory dumping [156]. Sandostatin LAR (10 mg/4 weeks intramuscularly for 6 months) was as effective as octreotide in ameliorating severe dumping symptoms, and it was more effective than octreotide in increasing body weight and improving quality of life [157].

The efficacy and safety of pasireotide in late dumping syndrome was evaluated in a single-arm, multicenter, intrapatient dose-escalation, phase II study, which included 43 patients with a history of bariatric surgery or upper gastrointestinal cancer surgery. The proportion of patients without hypoglycemia was 60.5% (26 of 43 patients), 36.4% (12 of 33 patients), and 39.4% (13 of 33 patients) at the end of the pasireotide SC phase (month 3), at the end of pasireotide IM phase (month 6), and at the end of extension phase (month 12), respectively. In addition, pasireotide was associated with an improvement in health-related QoL measured via the 36-Item Short Form Health Survey and the symptoms of both early and late dumping syndrome [158].

#### 5.3.5. Digestive and Lymphatic Fistulas

Fistulas are a major complication of surgery and are associated with high morbidity and mortality (10–35%) [159]. The most proximal in the intestine (esophagus, stomach, duodenum, and jejunum) and those that have a high debit (hydroelectrolytic losses) are fistulas of difficult management. SSAs are useful in the treatment of fistulas due to their ability to inhibit digestive secretions (salivary, gastric, intestinal, pancreatic, and biliary) and decrease digestive peristalsis. It is also useful in lymphatic fistulas by reducing thoracic duct lymph flow rate. Several studies have shown the benefit of octreotide and lanreotide, achieving a higher fistula closure rate. With respect to the mortality rate, the results of the studies are contradictory. Most of these studies agree that the greatest benefit occurs in the first 10 days of treatment. Therefore, SSAs are an effective adjuvant treatment in the management of fistulas that do not respond to conventional treatment with intestinal rest and total parenteral nutrition. It is also used prophylactically in pancreatic surgery [160,161,162,163].

#### 5.3.6. Acute Bleeding from the Gastrointestinal Tract

Blood vessels express SSTR2. SSAs at therapeutic doses exert a direct vasoconstrictor effect through the potentiation of the effect of other vasoconstrictor agents and indirectly by inhibiting the release of vasodilator peptides (mainly glucagon). The splanchnic vasoconstriction causes a decrease in the portal and portocolateral blood flow with the consequent reduction in portal venous and intravaricose pressure [164].

In a meta-analysis that included seven studies (*n* = 301 patients) that compared the efficacy of SSAs with vasopressin in the treatment of acute bleeding from esophageal varices, it was observed that the initial control of bleeding was better and the incidence of adverse effects was lower with SSAs, but the incidence of early recurrence was lower with vasopressin [165]. Another meta-analysis that included three studies (*n* = 302 patients) that compared the efficacy of SSAs with Terlipressin found no statistically significant differences in the control of acute bleeding, the incidence of early recurrence and adverse events, transfusion requirements, or survival [166]. Some meta-analysis found that octreotide provided better bleeding control than vasopressin/terlipressin [167] and terlipressin [168] in patients with acute esophageal variceal bleeding.

There is no difference between vasopressin/terlipressin and SSAs in the prevention of re-bleeding after the initial treatment of bleeding esophageal varices [169]. Treatment with SSAs should be started as soon as possible and in conjunction with endoscopic treatment. 

#### 5.3.7. Hepatorenal Polycystosis

Polycystic kidney disease, with or without polycystic liver disease, is an autosomal dominant inheritance condition (*PKD1* gen) characterized by renal cysts, liver cysts, and cerebral aneurysm. Cyst formation is mediated by cAMP and adenylyl cyclase agonists because both stimulate the proliferation of epithelial cells of the kidneys [170]. By preventing the formation and growth of cysts, it preserves the original tubular architecture and, therefore, prevents deterioration of renal function. SSAs act on the SSTR2, antagonizing the activity of adenylyl cyclase and, therefore, cAMP, in kidney and liver cells [170]. 

In vitro and in vivo studies have shown that octreotide achieves a reduction in renal and hepatic cysts with few side effects. SSAs have a good safety profile and can provide effective hepatoprotection and renoprotection [171]. However, long-term studies that include a larger number of patients are required. 

#### 5.3.8. Refractory Chronic Diarrhea

Refractory chronic diarrhea is defined as a diarrheal episode of more than 14 days that does not respond to specific antimicrobial therapy or nonspecific antidiarrheal therapeutic measures. The effects of SSAs that contribute to their possible antidiarrheal actions are the inhibition of gastrointestinal motility, exocrine digestive secretions, and intestinal absorption. However, the inhibition of pancreatic secretion could cause diarrhea due to fatty malabsorption.

Octreotide studies have been conducted for the treatment of Refractory chronic diarrhea. The most representative studies with octreotide for the control of AIDS/chemotherapy/short bowel syndrome and graft versus host disease-induced diarrhea have demonstrated a response rate between 41–100%, 74–96%, 16–72%, and 50–100%, respectively [172]. 

Probably, the use of octreotide for long periods of time is associated with a higher response rate. SSAs have also demonstrated a role in the control of refractory diarrhea associated with medullary thyroid cancer. In a patient with metastatic medullary thyroid carcinoma and refractory diarrhea, octreotide (100 µg three times a day subcutaneously), fecal incontinence control, and sustained diarrhea improvement were achieved [173].

#### 5.3.9. Non-Endocrine Tumors

Several in vivo and in vitro studies have shown antisecretory and antitumor effects of SST in cancer. The exact mechanism of the antitumor effect of SST is unknown, but some options include: inhibition of proliferation and induction of apoptosis (direct effects), decrease in gastrin, secretin, cholecystokinin (CCK), and IGF-1 levels directly by suppressing of *IGF-1* gene expression or indirectly through inhibition of hepatic production (indirect effects), and peritumoral vasoconstriction that causes a decrease in nutritional support [174]. However, the role and therapeutic potential of SSAs in non-endocrine tumors (breast, colon, prostate, lung, exocrine pancreatic, and hepatocellular) are currently unknown. 

The most frequent in women and the second most common cancer in the general population is breast cancer. Most breast cancers (50–70%) overexpress SSTR (mostly SSTR2) compared to normal tissues. The presence of SSTR2 is considered a favorable factor in breast cancer, associated with low invasion and proliferation. However, in breast cancers that express truncated variant SSTR5-TMD4, the beneficial function of SSTR2 is compromised. The prevalence of SSTR5-TMD4 mRNA in samples of breast cancers classified as G3 was detected at 28–49%, and their presence is related with the absence of immunoreactivity to p53, estrogen receptor and Her2neu, and lymph node metastases [175]. Therefore, SST5R-TMD4 expression increased malignancy features such as invasion and proliferation. Several studies suggest a significant role of SSAs in breast cancer; however, their full potential has not been revealed because they included a small number of patients and the results are contradictory. Although studies have shown limited success, it should be studied whether efficacy could be improved when combined with other antitumor therapies [176].

The most frequent tumor in older men is prostate cancer. In a rat prostate tumor model, lanreotide was as effective as castration, and therapeutic benefit was also observed in the hormone-resistance phase [177]. Lanreotide (30 mg/week by intramuscular injection) in 30 patients with prostate cancer refractory to metastatic hormones with an average duration of 12 weeks (2–60 weeks) improved functional status (40%) and bone pain (35%), decreased >50% in prostate-specific antigen (PSA) levels, and stabilized PSA levels in 20% and 16% of patients, respectively. An annual survival rate of 89% was achieved in the group of patients who responded to PSA levels and 64% in patients with progressive disease [178]. Another study in 10 patients with hormone-resistant metastatic disease with lanreotide plus ethinyl estradiol showed a >50% decrease in PSA levels in 90% of patients and palliation of bone pain in all patients [179]. 

Colon neoplasms express cyclooxygenase-2 (COX-2), a key enzyme in the production of prostaglandin E2, which contributes to cell proliferation. SSAs, by activating SSTR3 or SSTR5, modify the expression and function of COX-2 in colon cancer cells, inhibiting tumor cell proliferation [180]. One study showed that three out of four colon cancers were inhibited by SSA administration. Although the exact mechanism of this effect is unknown, it is speculated that a possible mechanism could be the indirect effect of SSAs on the secretion of gastrin or on the modulation of gastrin receptors present in colon cancers, as gastrin receptors were found to be downregulated in these tumors after treatment with SSAs. In vitro studies have shown that when SST is combined with gastrin, there is a decrease in cell proliferation of 34% in cell counts compared to gastrin alone [181].

Several studies suggest a possible role for SSAs in the adjuvant treatment of human small cell lung cancer (SCLC) that expresses SSTR (mainly SSTR2) [182]. In vitro studies have shown inhibition of tumor cell proliferation. In a study including 20 patients (six newly diagnosed and 14 relapsed disease), IGF-1 levels decreased significantly with octreotide (250 μg/day), but tumor burden did not change [183]. Another study reported a significantly better median survival and median time to progression in patients with SCLC in treatment with lanreotide 30 mg and chemotherapy compared to chemotherapy alone [184]. Moreover, SSAs have shown to successfully control the clinical symptoms of ectopic hormone secretion in bronchial carcinoids [185]. 

Regarding hepatocarcinomas, one trial in 58 patients with advanced hepatocellular carcinoma treated with octreotide (250 μg subcutaneously twice daily) vs. supportive care showed greater survival for octreotide (median survival of 13 months and 4 months, respectively) [186]. However, the HECTOR trial showed no survival benefit for hepatocellular carcinoma patients treated with Sandostatin LAR 30 mg intramuscularly every 4 weeks compared with placebo [187]. The variability of the results in survival in hepatocellular carcinoma could be explained by the individual differences of each patient and the variations of SSTR.

## 6. Adverse Events

SSAs are generally well tolerated. Adverse events may begin shortly after the first administration of the drug and may decrease progressively over the subsequent weeks as the treatment continues [188]. The most reported adverse events include injection site discomfort and erythema, gastrointestinal disturbances such as diarrhea, abdominal pain, nausea and vomiting, biliary sludge or gallstones, and modest and transient negative impact on glucose homeostasis. Cholelithiasis represents the most serious complication of SSAs, but is generally asymptomatic, and has been reported in 3–56% of patients. Pasireotide has shown a safety profile as expected for an SSA, except for the degree of hyperglycemia [189]. Uncommon side effects include sinus bradycardia, asthenia, headache, pruritus, decreased libido, increased serum bilirubin, and constipation [190]. Alopecia has also been reported [191]. High doses of SSAs have not shown differences in common adverse events when compared to standard doses [192]. 

## 7. Conclusions

SSAs are an invaluable therapeutic option in the diagnosis and treatment of somatotropinomas, thyrotropinomas, and functioning and non-functioning gastroenteropancreatic neuroendocrine tumors. They should also be considered an effective and safe therapeutic alternative of corticotropinomas, gonadotropinomas, and prolactinomas resistant to dopamine agonists. Somatostatin analogs have also shown to be useful in the treatment of other endocrine diseases (congenital hyperinsulinism, Graves’ orbitopathy, diabetic retinopathy, diabetic macular edema), non-endocrine neoplasms (breast, colon, prostate, lung, and hepatocellular), and digestive diseases (chronic refractory diarrhea, hepatorenal polycystosis, gastrointestinal hemorrhage, dumping syndrome, and intestinal fistula). 

## Figures and Tables

**Table 1:** Indications of medical treatment with SSAs.

**On-Label**
Endocrinological indications
(a) First generation SSAs: —Acromegaly * —Symptoms associated with functional GEP-NETs —Unresectable, well-or moderately-differentiated, locally advanced or metastatic GEP-NETs —Thyrotropinomas *
(b) Second generation SSAs: —Acromegaly * —Cushing’s disease *
Digestive indications
(a) First generation SSAs: —Prevention of complications after pancreatic surgery —Upper gastrointestinal hemorrhage due to gastroesophageal varices in patients with cirrhosis
**Off-label**
Endocrinological indications
—Gonadotropinomas * —DARPs —GEP-NETs in MEN-1 —Refractory diarrhea associated with medullary thyroid cancer —Congenital hyperinsulinism or persistent hyperinsulinemic hypoglycemia of infancy —Diabetic retinopathy and diabetic macular edema —Graves’ orbitopathy
Digestive indications
—Dumping syndrome —Digestive and lymphatic fistulas —Hepatorenal polycystosis —Refractory chronic diarrhea
Non-endocrinological tumors
—Breast cancer —Colon cancer —Prostate cancer —Small cell lung cancer —Exocrine pancreatic cancer —Hepatocellular cancer

* In those cases where surgery is not appropriate, after the failure of surgery and radiotherapy, or in the interim period until radiotherapy is completely effective. GEP-NETs: Gastroenteropancreatic neuroendocrine tumours. DARPs: Dopamine agonists resistant prolactinomas. MEN 1: Multiple endocrine neoplasia type 1.

## Abbreviations

SSAs	Somatostatin Analogs
SST	Somatostatin
SSTR	Somatostatin Receptor
IGF	Insulin-like Growth Factor
DAs	Dopamine Agonists
DARPs	Dopamine Agonist-resistant prolactinomas
NETs	Neuroendocrine Tumors
GEP-NETs	Gastroenteropancreatic neuroendocrine tumors
GH	Growth Hormone
LAR	Long-acting release
ATG	Autogel
PRL	Prolactin
ACTH	Corticotropin
TSH	Thyrotropin
MRI	Magnetic Resonance Image
ECL	Enterochromaffin-like
DR	Diabetic Retinopathy
DME	Diabetic macular edema
CHI	Congenital hyperinsulinism
PSA	Prostate-specific antigen
